# The complete genomic sequencing of a *Klebsiella pneumoniae* isolate derived from the blood of a patient suffering from severe neurological problems in Zhengzhou, China

**DOI:** 10.1128/mra.01290-23

**Published:** 2024-08-09

**Authors:** Huishuang Yang, Jun Zhang, Sayyed Salman, Weidong Zhu

**Affiliations:** 1 The Third Clinical Medical College of Zhejiang Chinese Medicine University, Hangzhou, China; 2 Collaborative Innovation Center for Diagnosis and Treatment of Infectious Diseases, State Key Laboratory for Diagnosis and Treatment of Infectious Diseases, the First Affiliated Hospital, College of Medicine, Zhejiang University, Hangzhou, Zhejiang, China; 3 Traditional Chinese Medical Hospital of Zhuji, Huandong, Zhuji, China; Indiana University, Bloomington, Bloomington, Indiana, USA

**Keywords:** *Klebsiella pneumoniae*, genomic sequencing

## Abstract

This study presents the comprehensive analysis of the genomic sequence of *Klebsiella pneumoniae* strain FAHZZU7042hy, having a 5,690,191 bp chromosome size. This strain was obtained from a blood sample of a patient suffering from severe neurological problems in Zhengzhou, China, 2023.

## ANNOUNCEMENT

This study analyzes the presence of *Klebsiella pneumoniae* in a female patient with severe neurological problems. A patient’s 5 mL blood sample was collected using the BacT/ALERT Microbial Detection System BPA Culture Bottle Biomerieux to detect bacteria presence. After confirmation, the sample was cultured on MacConkey agar with 2 mg/L meropenem at 37°C overnight. Colonies with distinct morphologies were repeatedly streaked on MacConkey agar treated with meropenem to isolate a pure culture. The pure isolates were then identified using matrix-assisted laser desorption/ionization time-of-flight mass spectrometry (Bruker, Bremen, Germany) (1, 2).

Following conformation, Genomic DNA was extracted from a culture grown overnight at 37°C on Mueller Hinton agar using the Qiagen Yeast/Bact kit (Gentra Puregene). The genomic DNA was then processed for Oxford Nanopore and Illumina sequencing. Fragmentation was performed using Diagenode Picorupto with sonication for 15 seconds and six cycles to achieve fragments of 350 base pairs (bp) for short-read sequencing. The sequencing library was prepared using the Nextera XT kit (Illumina, USA) and sequencing was conducted on the Illumina NovaSeq 6000 platform, employing a paired-end sequencing strategy with 150 bp reads. The A-tailed fragments were ligated with paired-end adaptors, and subjected to PCR amplification with a 500 bp insert. Post-PCR, the products were purified using the AMPure XP system (USA). The library’s quality was evaluated using the Agilent 5400 system (USA) and quantified by qPCR (1.5 nM) at machine’s default parameters except where otherwise noted. The quality statistical analysis of the short raw read was evaluated by Fastp (version 0.23.1). The DNA library was prepared for long reads using the Ligation Sequencing Kit (SQK-LSK114). Before library preparation, genomic DNA was not mechanically or enzymatically sheared for long-read sequencing by ONT flow cell (R9.4.1). The raw reads obtained from the PromethION sequencing platform were first evaluated using the MinKNOW software (version 23.07.12) to assess real-time quality control metrics. Subsequently, the reads were subjected to base calling using the Oxford Nanopore Technologies Guppy software (version 0.17.1). The Nanofilt tool (version 2.8.0) was employed to filter out reads with a mean Q-score lower than 10, thereby excluding them from further analysis.

The hybrid assembly was conducted via Unicycler (version 0.4.7) and employed TBLASTN to identify DnaA or RepA alleles in every finished replicon as the start gene (3). The sequence was then submitted to NCBI for annotation and bioinformatics analysis by NCBI Prokaryotic Genome Annotation Pipeline (6.6) (4, 5), and it was performed using a Best-placed reference protein set and GeneMarkS-2+ Acquired antibiotic resistance genes in L2890hy were identified by ResFinder 4.1. Related metrics areb listed in Table 1.

**TABLE 1:** Features of the complete whole-genome sequences of *K. pneumoniae* strain FAHZZU7042hy

Feature	Parameter	Value
Long read	Number of reads	105,517
	Total bases (bp)	1,083,979,383
	N50 read length (bp)	8,620
	Mean read quality	11.3
Short read	Total bases	10,002,456
	Raw base (G)	1.5
	Clean base (G)	1.5
	Q20 (%)	97.40
	Q30 (%)	92.76
	GC (%)	56.69
Assembly	Genome size (bp)	5,690,191
	Long read coverage (X)	180
	Short read coverage (X)	250
	GC content (%)	56.9
	N50 value	5,326,189
Plasmid1 (GI: 2623820905)	Sequence size (bp)	201,712
	GC content (%)	52.7
Plasmid2 (GI: 2623820907)	Sequence size (bp)	108,423
	GC content (%)	49.5
Plasmid3 (GI: 2623820908)	Sequence size (bp)	41,711
	GC content (%)	39.5
Plasmid4 (GI: 2623820910)	Sequence size (bp)	12,156
	GC content (%)	38.5
	Resistance gene (antimicrobial)	
	Chromosome	*aac()*-IId [EU022314] (aminoglycoside)
		*fosA* [KU254579] (fosfomycin)
		*oqxA* [EU370913], *oqxB* [EU370913] (quinolone)
		*blaCTX*-*M*-*15* [AY044436], *blaSHV-28* [AF299299] (beta-lactam)
	Plasmid1	*aph(3')-Ia* [V00359], *aac(6')-Ib-cr* [DQ303918] (aminoglycoside)
		*blaCTX-M-15* [AY044436], *blaOXA-1* [HQ170510], *blaTEM-1B* [AY458016], *blaKPC-2* [AY034847] (beta-lactam)
		*catB3* [AJ009818] (amphenicol)

The complete and circular chromosome size of *K. pneumoniae* FAHZZU7042hy is 5,690,191 bp. The GC content was approximately 56.9%. Antimicrobial resistance genes analysis showed that the chromosome contained two antimicrobial resistance genes, *blaCTX-M-15* and *blaSHV-28* (beta-lactam), including the *aac(3)-IId* (aminoglycoside) and *oqxA* and *oqxB* (quinolone). The PlasmidFinder analyses showed that *K. pneumoniae* has six antibiotic resistance genes on the plasmid, along with the *blaCTX-M-15, blaOXA-1, blaTEM-1B,* and *blaKPC-2* (beta-lactam) genes.

## Data Availability

The whole-genome sequence of *K. pneumoniae* strain FAHZZU7042hy has been deposited in GenBank under BioProject accession number PRJNA1037684, BioSample accession number SAMN38195470, Nanopore SRA number SRR26780951, and Illumina SRA number SRR27293095. The version described in this paper is the first version.
